# Supplementary material for: Identifying regions of importance in wall-bounded turbulence through explainable deep learning
Source: Nat Commun. 2024 May 13;15:3864. doi: 10.1038/s41467-024-47954-6 (PMC11091079; doi:10.1038/s41467-024-47954-6)
Supplement: Supplementary file 1 — Supplementary Information [file 41467_2024_47954_MOESM1_ESM.pdf]

nature portfolio

Peer Review File

Identifying regions of importance in wall-bounded turbulence through explainable deep learning

Open Access This file is licensed under a Creative Commons Attribution 4.0 International License, which permits use, sharing, adaptation, distribution and reproduction in any medium or format, as long as you give appropriate credit to the original author(s) and the source, provide a link to the Creative Commons license, and indicate if changes were made. In the cases where the authors are anonymous, such as is the case for the reports of anonymous peer reviewers, author attribution should be to 'Anonymous Referee' followed by a clear attribution to the source work. The images or other third party material in this file are included in the article's Creative Commons license, unless indicated otherwise in a credit line to the material. If material is not included in the article's Creative Commons license and your intended use is not permitted by statutory regulation or exceeds the permitted use, you will need to obtain permission directly from the copyright holder. To view a copy of this license, visit http://creativecommons.org/licenses/by/4.0/.

REVIEWER COMMENTS

Reviewer #1 (Remarks to the Author):

The paper titled "Explaining wall-bounded turbulence through deep learning" by authors Cremades et al. explores the use of Shapely additive explanations for an explainable deep-learning method for turbulent wall-bounded flows. According to the authors, the paper applies the method to Reynolds-stress structures in wall turbulence to quantify the connection between these structures and the dynamics of the flow.

I have a few main concerns regarding the paper. First is the use of CNNs in the work. It seems as if the only use for CNN is to predict the next time step such that the SHAP can be applied to the consecutive snapshots. However, considering the small Reynolds number, the resources required to train the CNN, the error in the predictions, and many more possible issues, I do not see why the authors are using the CNN. A single-step time integration is cheap and easy to perform--without any concern for additional errors due to NN-based predictions. The SHAP can be applied to these consecutive snapshots. It feels like the use of ML for the sake of using ML. Unless there are significant time savings (which I doubt due to the low Reynolds number and small simulation domain) or there is a particular reason behind using the CNN, I think the authors should revisit this choice.

Secondly, the SHAP values are computed as a linear regression of the input features. In a chaotic nonlinear process, I wonder how much the SHAP values can really explain the flow.

The final concern I have is regarding the interpretation of the SHAP values. The authors put heavy emphasis on this being an explainable deep-learning method. However, the authors already choose the relevant structures as the Reynolds stress events. The additional analysis is done through similar methods as previous studies--looking at aspect ratios of highlighted structures, the energy contained, etc. I have a hard time finding this method to be particularly "explainable". Furthermore, I don't see how the conclusions drawn can explain the "connection between the Reynolds-stress structures and the dynamics of the flow" as the authors mention in their abstract.

Reviewer #3 (Remarks to the Author):

In this paper the authors explore the use of a trained convolutional neural network along with an explanatory metric (SHAP) to identify structures in a turbulent channel flow that are "important" to its

evolution. This is an interesting approach which may be useful to address an important issue in turbulence research of identifying the dynamical processes that are critical to its observed behavior. That said, there are several shortcomings of the manuscript in its current form and limitations of the study. These are outlined below:

1) The turbulent channel flow data used in this study is at exceptionally low Reynolds number, and obtained from a simulation in a relatively small spatial domain. As a consequence, the applicability of the results to flows of engineering interest, which are at much higher Reynolds number, is limited. Further, taken as an exploration of the utility of the analysis technique, the results are also limited in applicability since at the higher Reynolds number, there is generally much more going on, and the interaction of the turbulence with the wall is more complex. Whether this approach is useful in that case in not clear. I would imagine the limitation would be that a much

more extensive CNN predictor would be needed, as well as much more data to train it. I think the authors need to acknowledge these limitations and make a convincing case for the utility of the technique in turbulence at Reynolds number high enough to be interesting.

2) In a related issue, on page 8, the authors indicate that the domain size is large enough to "recover all the statistical information." This is almost certainly not true. For example two-point statistics with large spatial separations will necessarily by impacted by the domain size. The authors need to be precise about what they are claiming.

3) I believe the predictions being made with the CNN are of the velocity field at a later time, given the field at the current time, though this is not very clearly stated. Further, it is not clear how far forward in time is being predicted; there is a vague statement about prediction of the "next time step." Surly this cannot be the computational time step from the DNS as this would seem to make no sense, since in this case the changes in the field from one time to the next are very small and determined by the instantaneous time derivative. So, what is the prediction time horizon? Why was it chosen? Please be very clear about what prediction is being made, and justify why it is meaningful.

4) The whole rationale for this study is that the SHAP metric can help identify what is "important" to the turbulence evolution. So, I was disappointed that there was not more discussion of this metric, and what it actually means mathematically, so that we can understand the implications in the context of turbulence evolution. Please correct this shortcoming.

Reviewer 1

The authors would like to thank the Reviewer for their comments and examination of our work. The Reviewer's comments are reproduced below in black, with our responses following each comment in blue text. References to changes we made in the revised manuscript are indicated here by blue bold text, and are highlighted using the same style in the revised version of the manuscript. Additional changes were made in response to the other reviewers, and are also highlighted in the revised manuscript.

The authors have done a good job of improving the paper. I still have a few comments regarding the content.

We thank the reviewer for their positive assessment of the revision, and we hope that we could address the comments below to the reviewer's satisfaction.

1) The authors say that this is a "non-intrusive" assessment of the flow. However, I would suggest that the calculation of the SHAP value is indeed an "intrusive" assessment as it is comparing the flow as is v. the flow that is combined with a zero-perturbation flow field.

We thank the reviewer for this comment. Indeed, this is a special case where the method is intrusive on the surrogate model built through the neural network. We modified the text as follows:

… , and is intrusive on the surrogate model, rather than the original governing equations.

2) Related to the previous comment, I feel that the zero-perturbation flow field is such a change to the flow field. There are better ways to perhaps remove the strong Reynolds stresses that form in the regions of interest, but the zero-perturbation is such a departure from typical turbulence that I am not sure that calculating the SHAP values from these results would be a good metric. How about scaling down the existing perturbations so that it's below the threshold for being identified as a Q-event?

This is another very interesting comment by the reviewer. We tested this in two additional cases: one where, instead of replacing the structure with a region of zero fluctuations, we scaled down the u and v fluctuations such that they are 5\% below the threshold to be considered a Q event, while making $w=0$. In the second case, we scaled u and v as in the previous case, but left w unchanged. As can be seen in the results below, although the quantitative values are obviously changed, the qualitative trends and main results of this work are the same as those obtained when all the fluctuations are zero:

<u>Case with w=0</u>

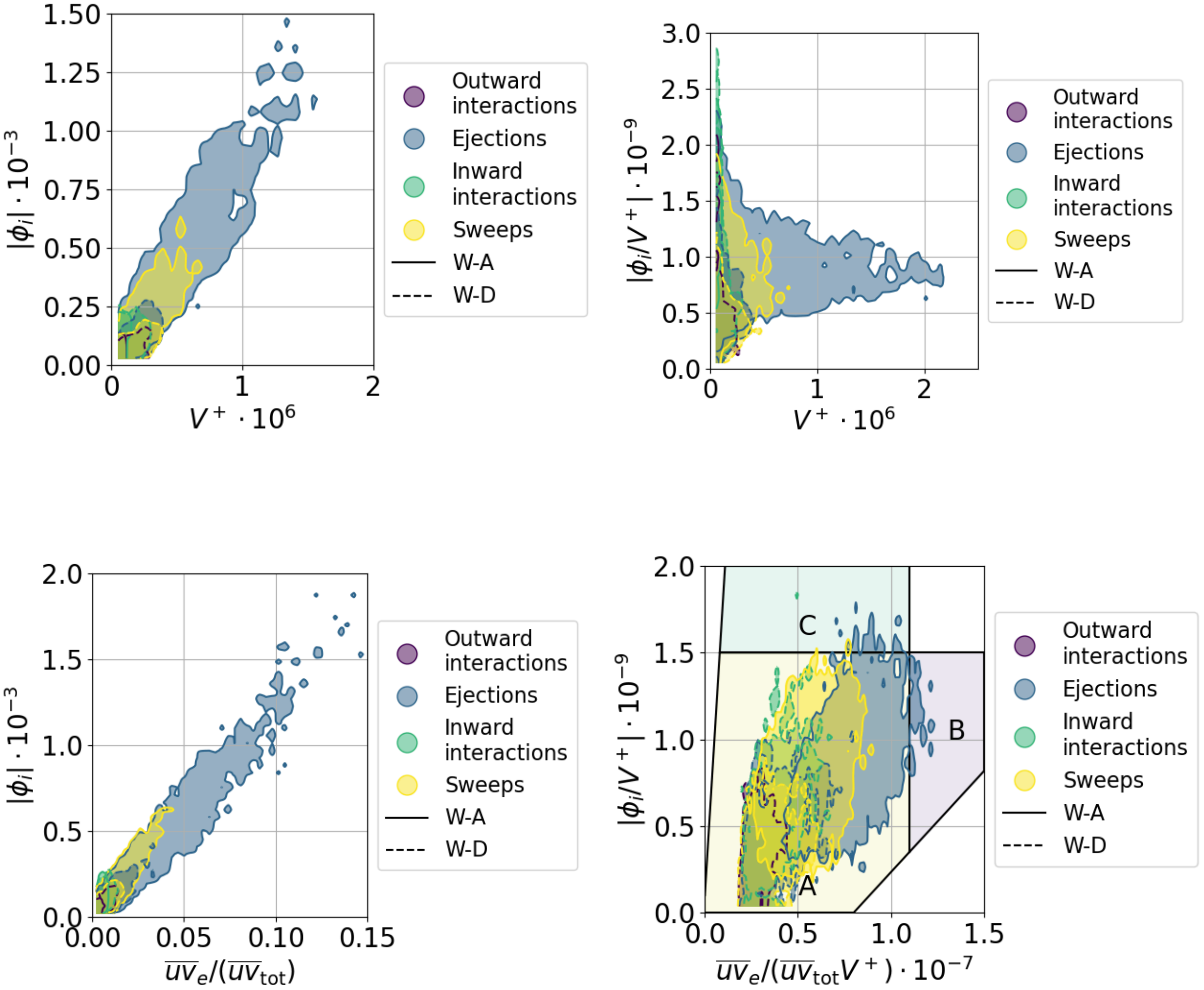

[Case with w unchanged]

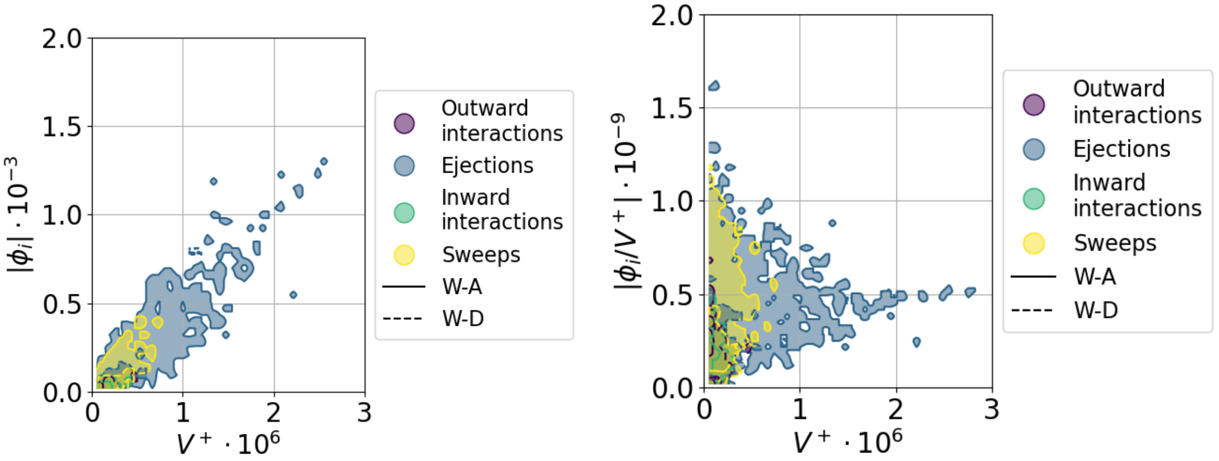

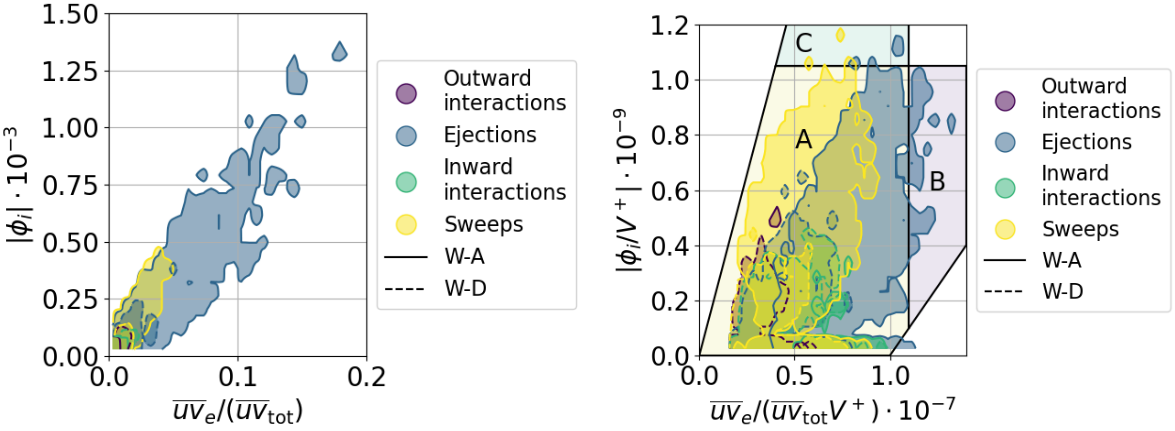

To reflect this, we added the following text to the Methods section:

Besides this, we also considered two additional cases: one where, instead of replacing the structure with a region of zero fluctuations, we scaled down the u and v fluctuations such that they are 5\% below the threshold to be considered a Q event, while making $w=0$. In the second case, we scaled u and v as in the previous case, but left w unchanged. In both cases the results and trends were qualitatively the same as when all the fluctuations are set to zero, and the main conclusions of this work were confirmed.

REVIEWER COMMENTS

Reviewer #1 (Remarks to the Author):

The authors have done a good job of improving the paper. I still have a few comments regarding the content.

1) The authors say that this is a "non-intrusive" assessment of the flow. However, I would suggest that the calculation of the SHAP value is indeed an "intrusive" assessment as it is comparing the flow as is v. the flow that is combined with a zero-perturbation flow field.

2) Related to the previous comment, I feel that the zero-perturbation flow field is such a change to the flow field. There are better ways to perhaps remove the strong Reynolds stresses that form in the regions of interest, but the zero-perturbation is such a departure from typical turbulence that I am not sure that calculating the SHAP values from these results would be a good metric. How about scaling down the existing perturbations so that it's below the threshold for being identified as a Q-event?

Reviewer #1 (Remarks on code availability):

I have looked a the code and it performs as described.

Reviewer #3 (Remarks to the Author):

The authors have exceeded my expectations regarding addressing my comments on the original manuscript. In particular, the application of the proposed techniques to experimental data is most welcome and, I believe, greatly improves the paper.

The revised manuscript addresses all of my concerns. I recommend the paper for publication.

Reviewer #3 (Remarks on code availability):

I inspected the code repository, and it appears that all relevant elements are present. However, I did not attempt to install or execute the code.

Reviewer 1

The authors would like to thank the Reviewer for their comments and examination of our work. The Reviewer's comments are reproduced below in black, with our responses following each comment in blue text. References to changes we made in the revised manuscript are indicated here by blue bold text, and are highlighted using the same style in the revised version of the manuscript. Additional changes were made in response to the other reviewers, and are also highlighted in the revised manuscript.

The authors have done a good job of improving the paper. I still have a few comments regarding the content.

We thank the reviewer for their positive assessment of the revision, and we hope that we could address the comments below to the reviewer's satisfaction.

1) The authors say that this is a "non-intrusive" assessment of the flow. However, I would suggest that the calculation of the SHAP value is indeed an "intrusive" assessment as it is comparing the flow as is v. the flow that is combined with a zero-perturbation flow field.

We thank the reviewer for this comment. Indeed, this is a special case where the method is intrusive on the surrogate model built through the neural network. We modified the text as follows:

… , and is intrusive on the surrogate model, rather than the original governing equations.

2) Related to the previous comment, I feel that the zero-perturbation flow field is such a change to the flow field. There are better ways to perhaps remove the strong Reynolds stresses that form in the regions of interest, but the zero-perturbation is such a departure from typical turbulence that I am not sure that calculating the SHAP values from these results would be a good metric. How about scaling down the existing perturbations so that it's below the threshold for being identified as a Q-event?

This is another very interesting comment by the reviewer. We tested this in two additional cases: one where, instead of replacing the structure with a region of zero fluctuations, we scaled down the u and v fluctuations such that they are 5\% below the threshold to be considered a Q event, while making $w=0$. In the second case, we scaled u and v as in the previous case, but left w unchanged. As can be seen in the results below, although the quantitative values are obviously changed, the qualitative trends and main results of this work are the same as those obtained when all the fluctuations are zero:

Case with w=0

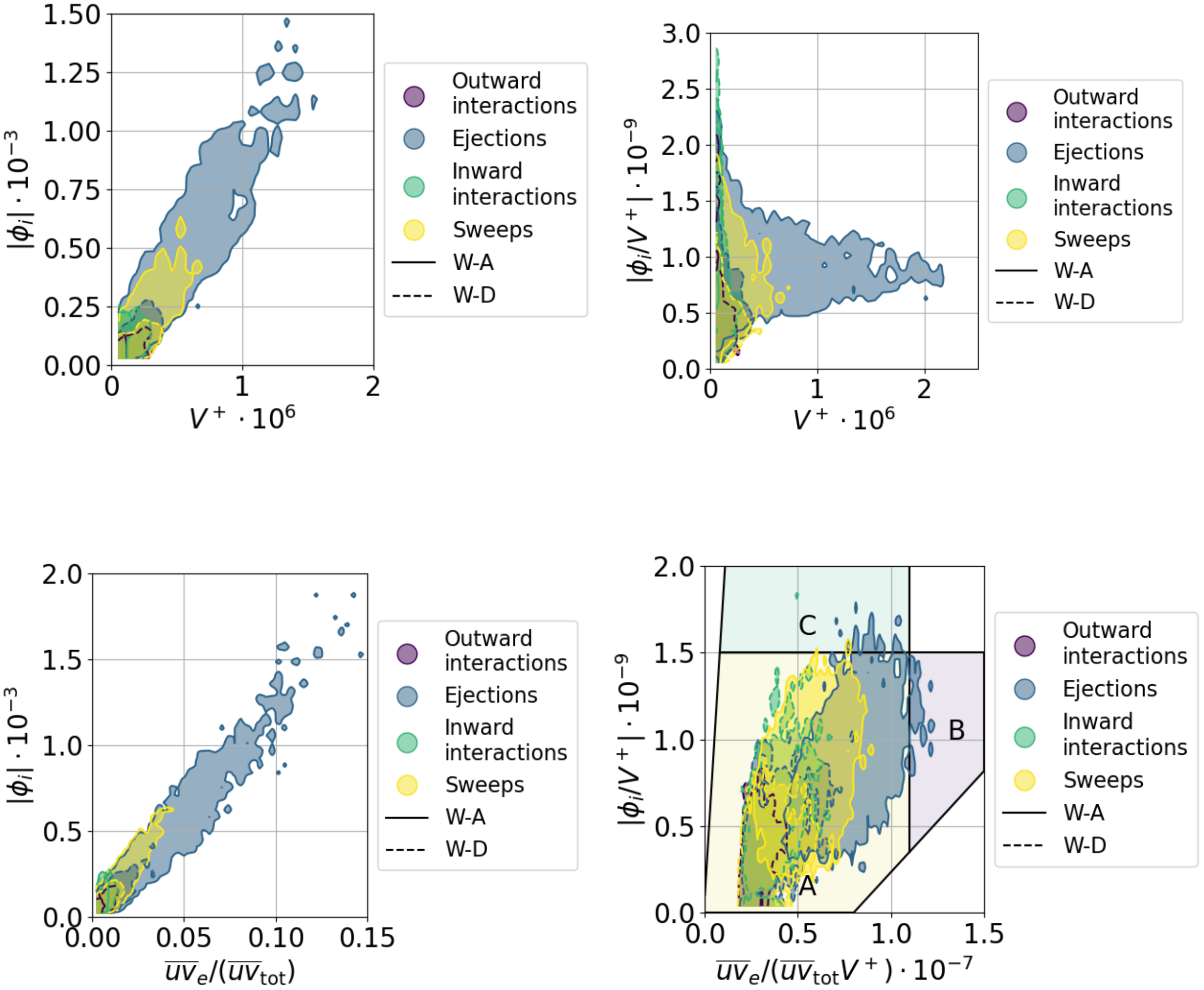

Case with w unchanged

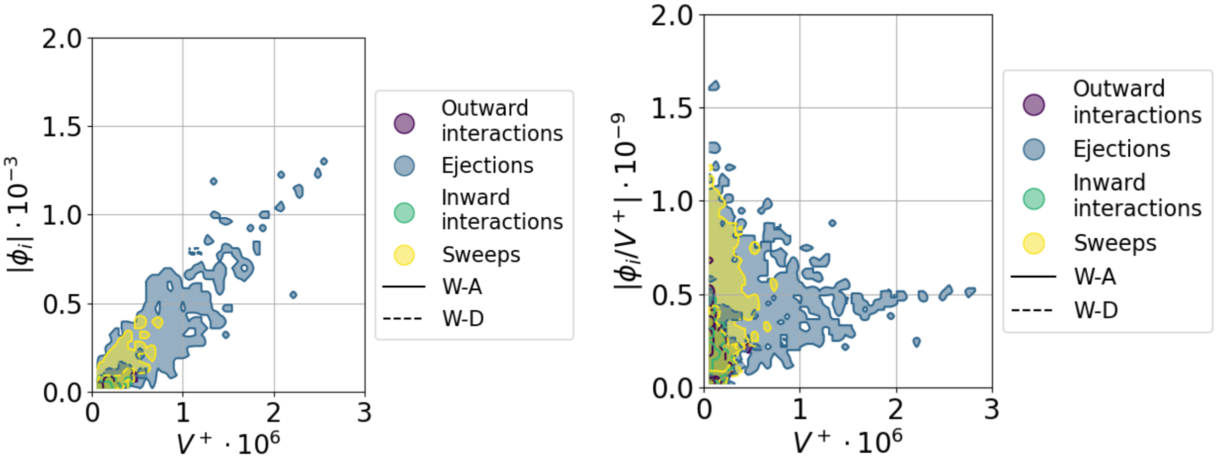

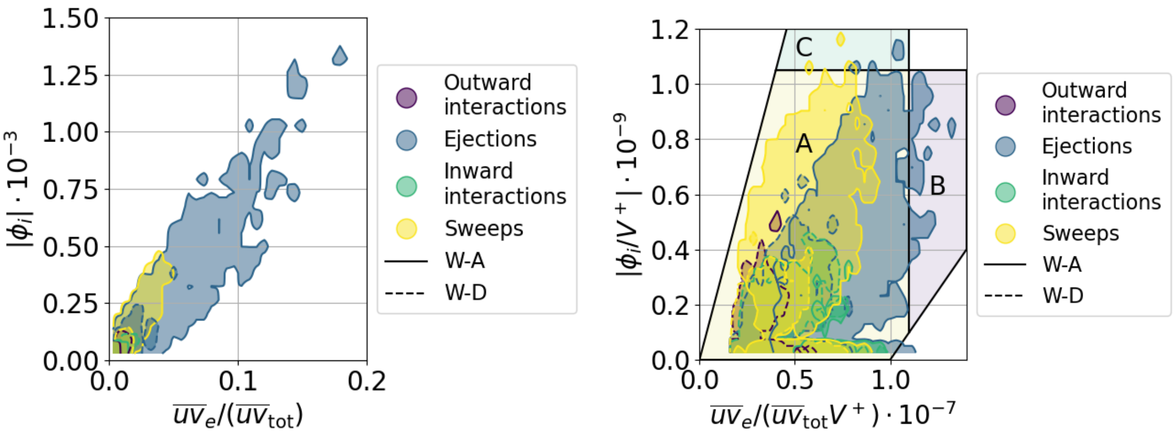

To reflect this, we added the following text to the Methods section:

Besides this, we also considered two additional cases: one where, instead of replacing the structure with a region of zero fluctuations, we scaled down the u and v fluctuations such that they are 5\% below the threshold to be considered a Q event, while making $w=0$. In the second case, we scaled u and v as in the previous case, but left w unchanged. In both cases the results and trends were qualitatively the same as when all the fluctuations are set to zero, and the main conclusions of this work were confirmed.

REVIEWERS' COMMENTS

Reviewer #1 (Remarks to the Author):

I am happy with the changes that the authors implemented. I recommend the paper for publication.
