# Supplementary material for: Identifying regions of importance in wall-bounded turbulence through explainable deep learning
Source: Nat Commun. 2024 May 13;15:3864. doi: 10.1038/s41467-024-47954-6 (PMC11091079; doi:10.1038/s41467-024-47954-6)
Supplement: Supplementary file 2 — Peer Review File [file 41467_2024_47954_MOESM2_ESM.pdf]

# Supplementary Material:
## Identifying regions of importance in wall-bounded turbulence through explainable deep learning

Andrés Cremades[1,2*], Sergio Hoyas[3], Rahul Deshpande[4], Pedro Quintero[2], Martin Lellep[5], Will Junghoon Lee[4], Jason P. Monty[4], Nicholas Hutchins[4], Moritz Linkmann[6], Ivan Marusic[4], Ricardo Vinuesa[1*]

[1*]FLOW, Engineering Mechanics, KTH Royal Institute of Technology, Stockholm, SE-100 44, Sweden.
[2]CMT-Motores Térmicos, Universitat Politècnica de València, Camino de Vera s/n, Valencia, 46022, Spain.
[3]Instituto Universitario de Matemática Pura y Aplicada, Universitat Politècnica de València, Valencia, 46022, Spain.
[4]Department of Mechanical Engineering, University of Melbourne, Parkville, Melbourne, 3010, VIC, Australia.
[5]SUPA, School of Physics and Astronomy, The University of Edinburgh, James Clerk Maxwell Building, Peter Guthrie Tait Road, Edinburgh, EH9 3FD, UK.
[6]School of Mathematics and Maxwell Institute for Mathematical Sciences, University of Edinburgh, Edinburgh, EH9 3FD, UK.

*Corresponding author(s). E-mail(s): andrescb@kth.se; rvinuesa@mech.kth.se;

# Wall-attached and wall-detached distributions

In this section we reproduce the results in Figures 4 and 5 from the main paper, distinguishing between wall-attached and wall-detached structures. Supplementary Figure 1 here is an extension of Figure 4 from the paper and Supplementary Figure 2 here is an extension of Figure 5 from the paper.

The results of Supplementary Figures 1 and 2 evidence the higher volume and importance of the large wall-attached ejections and sweeps, which transport most of the Reynolds stress [1, 2]. Furthermore, when the importance per unit volume is analyzed, the most important structures are small wall-attached ejections. For the largest structures, the SHAP value per unit volume tends to be moderate.

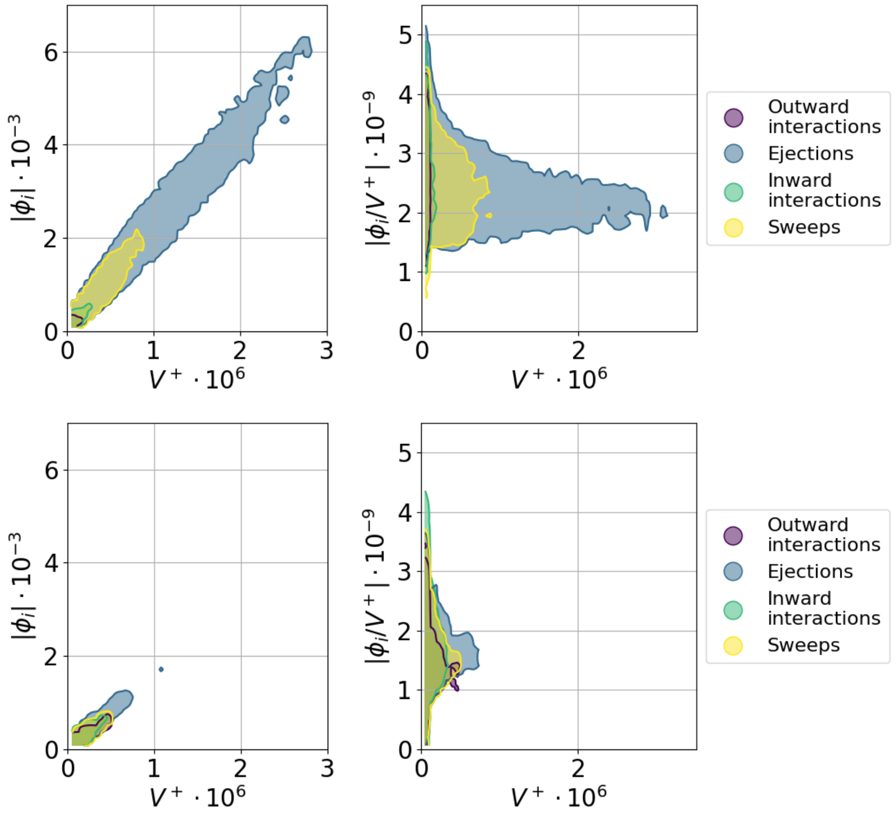


**Supplementary Figure 1: Magnitude of the SHAP values (left) and SHAP values per unit of volume (right) of the structures for different turbulent events as a function of their volume, expressed in inner units.** We show (top) wall-attached and (bottom) wall-detached structures. Note that the wall-normal distance $y^+ = 20$ is used to separate both types of structures. Extended version of Figure 4 in the main paper.

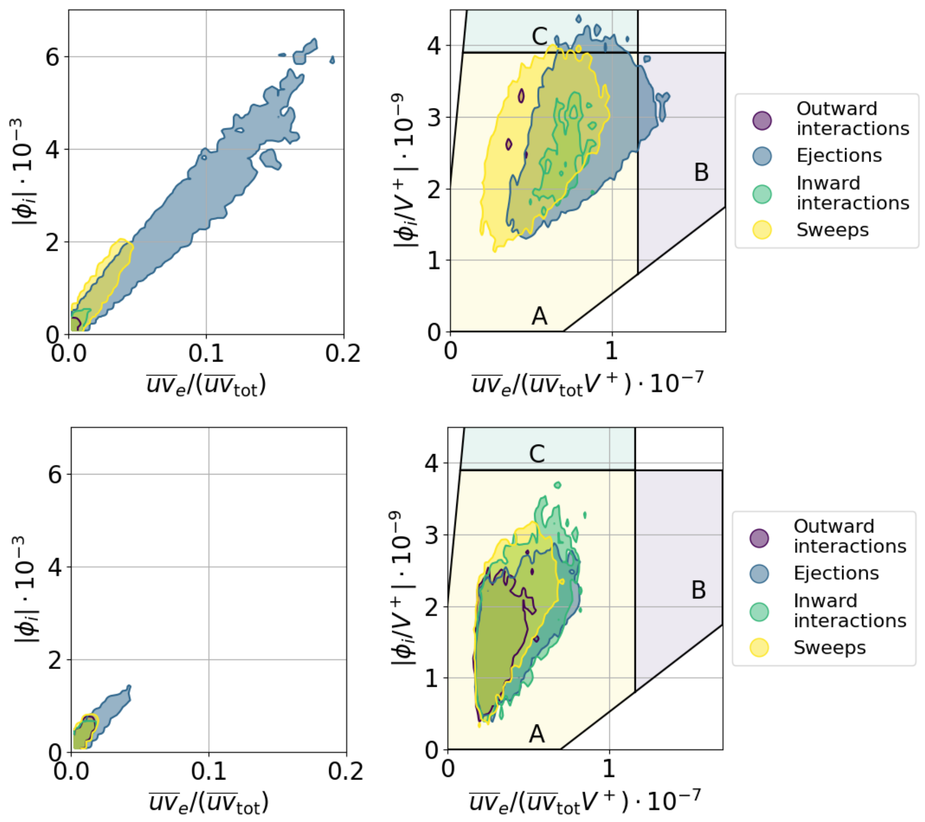


**Supplementary Figure 2**: **Magnitude of the SHAP values as a function of the fractional contribution to the total Reynolds shear stress (left) and same quantities scaled with the structure volume (right).** We show (top) wall-attached and (bottom) wall-detached structures. Note that the wall-normal distance $y^+ = 20$ is used to separate both types of structures. Extended version of Figure 5 in the main paper.

Regarding the relationship between the SHAP value and the tangential Reynolds stress, wall-attached ejections and sweeps transport most of the $\overline{uv}$, and thus they exhibit higher SHAP values. In addition, the wall-attached structures, mainly ejections and sweeps, exhibit higher Reynolds stress and SHAP values per volume unit. However, the structures with the highest SHAP values, located in zone C, differ from those with the highest Reynolds stresses, which are in zone B. Regarding the detached structures, they are smaller and, therefore, associated with lower SHAP values and $\overline{uv}$, while exhibiting medium and low SHAP values when scaling with the volume.

## Shape of the most relevant structures

Concerning the shape of the dominant structures, Supplementary Figure 3 shows the SHAP values of ejections and sweeps as a function of the geometrical aspect ratios of the structures: $\Delta x/\Delta y$ and $\Delta z/\Delta y$. Although the importance per unit volume of the outward and inward interactions is not negligible, they are not analyzed due to their comparatively smaller SHAP values. The aspect ratios are measured through the bounding box of each structure, where $\Delta x$ (streamwise), $\Delta y$ (wall-normal) and $\Delta z$ (spanwise) are its dimensions. As mentioned above, the most important structures are large wall-attached ejections. These structures are expected to exhibit the following geometric relations: $\Delta x \approx 3\Delta y$ and $\Delta z \approx \Delta y$ [1]. Although these results were obtained for a higher Reynolds number, the present methodology yields similar results ($3\Delta y < \Delta x < 6\Delta y$) for ejections and sweeps. Note that these structures are elongated in the streamwise direction [3]. In addition, the spanwise length of the structures is similar to their wall-normal size: $\Delta z \approx \Delta y$. Furthermore, the Q events with highest SHAP value exhibit a relationship between the spanwise and wall-normal directions close to $\Delta z \approx 1.5\Delta y$, a result in agreement with the literature [4]. Note that the isotropic structures ($\Delta x \approx \Delta y$) have much lower importance due to their small size. Furthermore, the most significant structures per unit volume are elongated in the streamwise direction, and the wall-normal direction is similar to the spanwise direction.

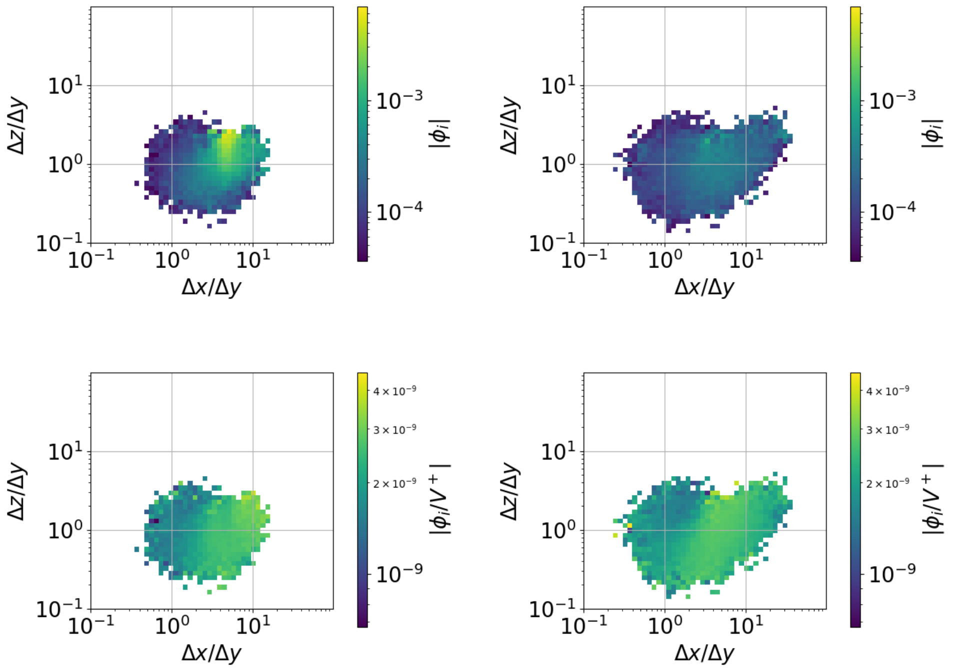


**Supplementary Figure 3: SHAP values (top) and SHAP values per unit volume (bottom) of the simulated data for the ejections (left) and the sweeps (right), where we show the aspect ratio of the boxes circumscribing the Reynolds-stress structures.** Note that the most important structures are generally located below the $\Delta z/\Delta y = \Delta x/\Delta y$ diagonal. These structures exhibit values of $\Delta y$ and $\Delta z$ lower than those of $\Delta x$, *i.e.* they are elongated in the streamwise direction.

The shape of the structures is also analyzed for the two-dimensional experimental data [5]. In this case, the aspect ratio of all the structure types is presented in Supplementary Figure 4. The results show that the ejections and the sweeps exhibit higher importance for aspect ratios of approximately $\Delta x/\Delta y \approx 3$. Furthermore, the SHAP per unit surface shows that the ejections are the most important, and in all the Q events the highest importance is associated with structures elongated in the streamwise direction. These results perfectly agree with the results from the numerical data, showing the consistency of the method.

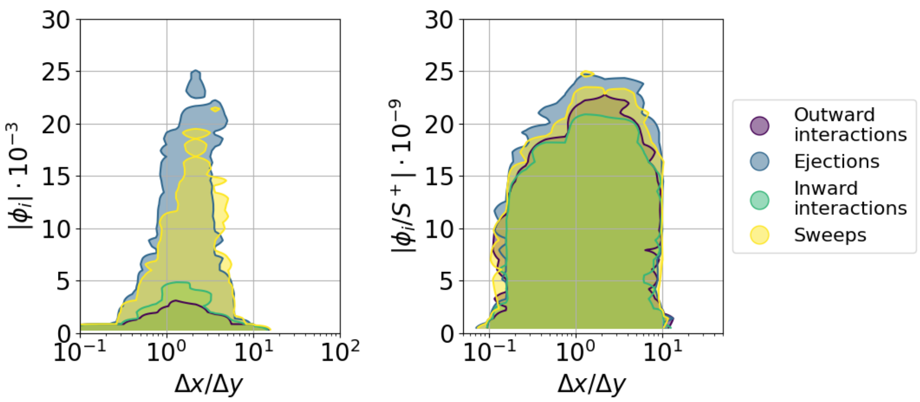


**Supplementary Figure 4**: **SHAP values (left) and SHAP values per unit surface (right) of the experimental data [5] as a function of the structure aspect ratio.** Note that the most important structures exhibit values of $\Delta x/\Delta y$ higher than 1, so they are elongated in the streamwise direction.

# Importance of the background

Finally, we note that the points not belonging to any structure are irrelevant for the energy transfer [1, 6]. To show this, every point not belonging to any Q event was merged into a single background structure. The SHAP per unit of volume of this background structure is negligible compared with the Q events.

# Supplementary References

[1] Lozano-Durán, A., Flores, O., Jiménez, J.: The three-dimensional structure of momentum transfer in turbulent channels. Journal of Fluid Mechanics **694**, 100–130 (2012)

[2] Jiménez, J.: Coherent structures in wall-bounded turbulence. Journal of Fluid Mechanics **842**, 1 (2018)

[3] Dong, S., Lozano-Durán, A., Sekimoto, A., Jiménez, J.: Coherent structures in statistically stationary homogeneous shear turbulence. Journal of Fluid Mechanics **816**, 167–208 (2017) https://doi.org/10.1017/jfm.2017.78

[4] Álamo, J.C., Jiménez, J., Zandonade, P., Moser, R.: Self-similar vortex clusters in the turbulent logarythmic region. Journal of Fluid Mechanics **561**, 329–358 (2006)

[5] Lee, J.H., Hutchins, N., Monty, J.P., Kozul, M.: Formation and evolution of shear layers in a developing turbulent boundary layer. Proc. 11th International Symposium on Turbulence and Shear Flow Phenomena (TSFP11) Southampton, UK, July 30 to August 2 (2019)

[6] Ganapathisubramani, B., Longmire, E.K., Marusic, I.: Characteristics of vortex packets in turbulent boundary layers. Journal of Fluid Mechanics **478**, 35–46 (2003)
